# Encouraging Volitional Pedaling in Functional Electrical Stimulation-Assisted Cycling Using Barrier Functions

**DOI:** 10.3389/frobt.2021.742986

**Published:** 2021-11-24

**Authors:** Axton Isaly, Brendon C. Allen, Ricardo G. Sanfelice, Warren E. Dixon

**Affiliations:** ^1^ Department of Mechanical and Aerospace Engineering, University of Florida, Gainesville, FL, United States; ^2^ Department of Electrical and Computer Engineering, University of California, Santa Cruz, Santa Cruz, CA, United States

**Keywords:** functional electrical stimulation (FES, ) cycling, barrier function, safety-critical, euler-Lagrange, control design

## Abstract

Stationary motorized cycling assisted by functional electrical stimulation (FES) is a popular therapy for people with movement impairments. Maximizing volitional contributions from the rider of the cycle can lead to long-term benefits like increased muscular strength and cardiovascular endurance. This paper develops a combined motor and FES control system that tasks the rider with maintaining their cadence near a target point using their own volition, while assistance or resistance is applied gradually as their cadence approaches the lower or upper boundary, respectively, of a user-defined safe range. Safety-ensuring barrier functions are used to guarantee that the rider’s cadence is constrained to the safe range, while minimal assistance is provided within the range to maximize effort by the rider. FES stimulation is applied before electric motor assistance to further increase power output from the rider. To account for uncertain dynamics, barrier function methods are combined with robust control tools from Lyapunov theory to develop controllers that guarantee safety in the worst-case. Because of the intermittent nature of FES stimulation, the closed-loop system is modeled as a hybrid system to certify that the set of states for which the cadence is in the safe range is asymptotically stable. The performance of the developed control method is demonstrated experimentally on five participants. The barrier function controller constrained the riders’ cadence in a range of 50 ± 5 RPM with an average cadence standard deviation of 1.4 RPM for a protocol where cadence with minimal variance was prioritized and used minimal assistance from the motor (4.1% of trial duration) in a separate protocol where power output from the rider was prioritized.

## 1 Introduction

Stationary cycling assisted by functional electrical stimulation (FES) can lead to long-term benefits for people with movement impairments due to neurological conditions such as stroke, spinal cord injury, traumatic brain injury, cerebral palsy, multiple sclerosis, and others Johnston et al. (2008); Ferrante et al. (2008); Hooker et al. (1992); Janssen et al. (2008); Trevisi et al. (2012). Individuals with neurological conditions can exhibit varying degrees of muscle control. For people with little to no volitional control, the FES cycling therapy must be supported by an electric motor, which provides additional torque about the pedal crank to maintain a beneficial cadence, as in studies such as Cousin et al. (2020); Hooker et al. (1992); Bellman et al. (2017); Duenas et al. (2020); Trevisi et al. (2012). When possible, electric motor support should be minimized in lieu of torque produced by the rider’s muscles via either FES or their own volition, which leads to higher intensity training by increasing the rider’s heart rate and oxygen uptake Hooker et al. (1992). Higher intensity training is a key factor in attaining long-term outcomes like increased muscular strength, cardiovascular endurance, bone mineral density, and caloric consumption Ouellette et al. (2004); MacKay-Lyons and Makrides (2002); Mohr et al. (1997). In the rehabilitation literature outside of cycling, various assist-as-needed approaches such as Asl et al. (2020); Dao and Yamamoto (2018); Pehlivan et al. (2015); Ding et al. (2014) encourage volitional contributions from the user. Relatively few works have investigated FES- and motor-assisted cycling programs where the primary objective is to encourage volitional contributions Harrington et al. (2012); Rouse et al. (2020); Johnston and Wainwright (2011). The objective of this work is to design controllers for both the electric motor and FES stimulation that facilitate volitional cycling by minimizing machine assistance while ensuring that the rider’s cadence is constrained to a user-defined range.

The kinematics of the rider’s legs during stationary cycling are such that applying FES to their muscles produces non-negligible torque only in certain regions of the crank cycle. To maximize torque production, stimulation patterns often feature discontinuous jumps triggered as a function of the crank angle by discrete logic variables. The interaction of the resulting continuous-time and discrete dynamics results in a hybrid control system. Barrier functions, or control barrier functions (CBF), can be used to design controllers for hybrid systems that ensure safety by rendering sets of states either forward invariant or asymptotically stable Ames et al. (2019); Maghenem and Sanfelice (2021); Glotfelter et al. (2017). This technique builds on ideas from the theory of control Lyapunov functions (CLF). CLFs are used to enforce particular constraints on the control input that result in a decrease in a Lyapunov function for states outside of the safe set Freeman and Kokotovic (1996); Sanfelice (2013). However, CLF-based approaches have typically not provided constructive methods for designing the control input at states in the safe set. Recent developments regarding CBFs have filled this gap by providing a systematic approach for extending the input constraints onto the safe set in a way that reduces the control effort on the interior of the set Ames et al. (2016). A popular approach for implementing CLF- or CBF-induced input constraints is with pointwise optimal control laws which, for certain classes of dynamics, take the form of quadratic programs (QP). Compared to past assist-as-needed control schemes, which have used methods such as deadzone functions Asl et al. (2020) or impedance control Ding et al. (2014), barrier functions can constrain the state within a broader class of safe sets. Moreover, the cost function in the accompanying pointwise optimal control law is customizable, leading to a range of possible controllers. Our preliminary work in Isaly et al. (2020) integrated zeroing CBFs with robust control tools from Lyapunov theory to synthesize a QP for an uncertain, continuous-time, motor-only cycling system. The controller in Isaly et al. (2020) constrains the rider’s cadence within a user-defined range while encouraging volitional pedaling by using minimal motor control effort. However, the more complex case where the rider is also stimulated by FES was not considered.

In this work, we extend the development of Isaly et al. (2020) to account for the hybrid dynamics introduced by adding FES stimulation. The resulting controller applies assistance based on the rider’s performance. FES assistance is only applied when the cadence cannot be maintained at a target value through volitional effort alone. Similarly, assistance from the electric motor is applied only when the combined FES and volitional efforts are insufficient. The controller accommodates a broad range of functional impairments and volitional ability by featuring customizable parameters, including nominal control inputs and tunable width of the safe range. Moreover, the rider’s safety is assured because the electric motor constrains the rider’s cadence to a uniformly globally asymptotically stable set through a continuous feedback controller. The continuity of the motor control law is an improvement upon the breakthrough strategy in Rouse et al. (2020) for encouraging volitional pedaling. In that work, no control effort was applied within a user-defined region, while the electric motor and FES were turned on discontinuously at the boundary of the region. Outside the region, assistive control effort switched discretely between FES and electric motor assistance to ensure that the electric motor did not prevent FES from inducing power output by the rider. In contrast, we decouple the motor and FES controllers and use more sophisticated design tools to develop a motor control law that is a continuous function of the cadence tracking error. The result is more comfortable training for the rider, while the staggered application of FES before motor effort still allows power output from the rider to be prioritized.

Experimental trials were performed on five able-bodied participants to demonstrate the effectiveness and versatility of the developed control system. The barrier function controller was shown to effectively constrain the cadence to a range of 50 ± 5 RPM for all but a negligible amount of time, and to outperform the controller in Rouse et al. (2020) and uncontrolled volitional pedaling for a protocol where minimal cadence variation was prioritized. The barrier function controller had a lower cadence standard deviation (Avg. 1.4 RPM) and constrained the cadence to a smaller range relative to the comparison cases, but generally produced more assistive torque from the motor than the controller in Rouse et al. (2020). To show how motor assistance can be reduced to prioritize power output from the rider, an alternative protocol was designed where the customizable parameters were configured with a wider safe range and a nominal amount of resistance from the motor. In the alternative trial, the motor was producing assistive torque for only 4.1% of the entire trial duration.

## 2 Dynamic Model

### 2.1 Hybrid Systems

The development in this work is based on the hybrid systems framework described in Goebel et al. (2012). A hybrid system $H=\left(C,F,D,G\right)$ with state $x\in X\subset R^{n}$ is modeled by

$$H:\left\{\begin{array}{cc} \dot{x}\in F\left(x\right)\; & x\in C\\ x^{+}\in G\left(x\right)\; & x\in D. \end{array}\right.$$ (1)

When the state is in the flow set $C\subset R^{n}$ , it is allowed to evolve continuously according to the set-valued flow map $F:R^{n}⇉R^{n}$ . When the state is in the jump set $D\subset R^{n}$ , it is allowed to change discretely according to the jump map $G:R^{n}⇉R^{n}$ . When x ∈ C ∩ D, either behavior is possible. The notion of a solution to H is defined precisely in Goebel et al. (2012), Def. 2.6. Briefly, a solution to H is a function $\left(t,j\right)\mapsto \phi \left(t,j\right)$ defined on a hybrid time domain $\text{dom}\;\phi \subset R_{\geq 0}\times N$ and is parameterized by the ordinary time variable $t\in R_{\geq 0}$ and the discrete jump variable j∈N . The set-valued mappings F and G map points in $R^{n}$ to subsets of $R^{n}$ so that, for example, the inclusion $x^{+}\in G\left(x\right)$ represents the fact that if a trajectory jumps from the state $\phi \left(t,j\right)$ , then its state $\phi \left(t,j+1\right)$ at the next discrete time instant is a point in the set $G\left(\phi \left(t,j\right)\right)$ .

Remark 1. Previous results (cf. Rouse et al. (2020); Bellman et al. (2017); Rouse et al. (2021)) have analyzed the dynamic model in the subsequent section using switched-systems tools. The decision to use a hybrid model here was motivated by the fact that forward invariance via barrier functions is not well characterized for switched systems, nor are many results available regarding the stability of noncompact sets. Hybrid systems can model broad classes of switched systems Goebel et al. (2012, Section 2.4).

### 2.2 Open-Loop Dynamics

Analogous to Eq. 1, one can also consider hybrid systems with inputs Sanfelice (2013). We use such a system to describe the control design but present our stability analysis in terms of a closed-loop system with the form in Eq. 1. The open-loop cycle-rider system is modeled as a continuous-time system $H_{u}=\left(C_{u},F_{u}\right)$ . Subsequently, discrete dynamics will be introduced due to the design of the controller. Adapting the model from our previous work in Bellman et al. (2017) and Isaly et al. (2020), the cycle’s Euler-Lagrange dynamics are modeled using the flow map

$$\dot{z}\in \left[\begin{array}{c} z_{2}\\ M^{-1}\left(z_{1}\right)\left[\tau_{u}\left(z,u\right)-\tau_{F}\left(z\right)\right] \end{array}\right]≜F_{u}\left(z,u\right),$$ (2)

and flow set $C_{u}≜R^{2}\times U$ . In (2), the state is $z\in R^{2}$ , where z_1_ denotes the cycle’s measurable crank angle, and z_2_ is the calculable angular velocity (equivalently, the rider’s cadence). The system has control inputs^1^ $u≜\left(u_{e},u_{M}\right)$ , where $u_{e}\in R$ is the current input to the cycle’s electric motor, and $u_{M}\in R^{6}$ is a vector of the electrical stimulation intensity inputs $u_{m}\in R$ , for each muscle $m\in M≜\left\{LQ,LG,LH,RQ,RG,RH\right\}$ . The elements of M indicate the quadriceps femoris (Q), gluteal (G), and hamstring (H) muscle groups for the left and right legs, respectively. The control inputs take values in the set $U≜R\times U_{M}$ , where $U_{M}≜{\left[0,\bar{u}\right]}^{6}\subset R^{6}$ indicates that the muscle control inputs are bounded by the constant $\bar{u}>0$ for the rider’s safety and comfort. The continuously differentiable function $M:R\to R_{>0}$ denotes the inertial forces from the cycle and rider’s legs. The set-valued mapping $\tau_{F}:R^{2}⇉R$ defines the dynamics of the system as

$$\tau_{F}\left(z\right)≜\tau_{b}\left(z_{2}\right)+V_{p}\left(z\right)z_{2}+G\left(z_{1}\right)+P\left(z\right)+T_{d}+T_{vol},$$ (3)

where $\tau_{b}:R\to R$ denotes the unknown torque due to viscous damping in the cycle, and $V_{p}:R^{2}\to R$ , G:R→R , and $P:R^{2}\to R$ are the unknown centripetal-Coriolis, gravitational, and passive viscoelastic tissue forces, respectively, applied by the combined human-cycle system. The aforementioned functions are continuous according to the dynamic models in Bellman et al. (2017) and Idsø (2002). According to the model in Bellman et al. (2017), the centripetal-Coriolis term is related to the mass and cadence by $V_{p}\left(z\right)=\frac{1}{2}\nabla M\left(z_{1}\right)z_{2}$ . In Eq. 3, $T_{vol}\subset R$ and $T_{d}\subset R$ are sets used to model all of the possible values of the rider’s volitional effort and other unknown disturbances, respectively^2^. The continuous function $\tau_{u}:R^{2}\times U\to R$ describes the torque produced by the control inputs and is defined as

$$\tau_{u}\left(z,u\right)≜c_{e}u_{e}+\tau_{FES}\left(z,u_{M}\right),$$

where c_e_ > 0 is the known electric motor control constant relating input current to output torque. The torque generated from FES inputs to the rider’s muscles $\tau_{FES}:R^{2}\times U_{M}\to R$ is given by

$$\tau_{FES}\left(z,u_{M}\right)≜\underset{m\in M}{\sum }g_{m}\left(z\right)u_{m},$$ (4)

where the continuous functions $g_{m}:R^{2}\to R$ denote the uncertain control effectiveness of each muscle. For each m∈M , let the closed set $Q_{m}\subset R$ denote the portion of the crank cycle when a particular muscle m is stimulated, which is selected based on a minimum threshold for the torque transfer ratio of each muscle group. In particular, there exist kinematic deadzones in the crank cycle where no muscle is able to produce useful torque Cousin et al. (2020).

The following properties of the cycle-rider system in Eq. 2 are derived from a detailed dynamic model, as discussed in Bellman et al. (2017).

Property 1. The inertial term is upper- and lower-bounded as ${\underline{c}}_{I}\leq M\left(z_{1}\right)\leq {\bar{c}}_{I}$ for all $z_{1}\in R$ , where ${\underline{c}}_{I},{\bar{c}}_{I}>0$ are known constants.

Property 2. The centripetal-Coriolis parameter is upper-bounded as $\left|V_{p}\left(z\right)\right|\leq c_{V}\left|z_{2}\right|$ for all $z\in R^{2}$ , where c_V_ > 0 is a known constant.

Property 3. The torque generated by gravity is upper-bounded as $\left|G\left(z_{1}\right)\right|\leq c_{G}$ for all $z_{1}\in R$ , where c_G_ > 0 is a known constant.

Property 4. The torque generated by the rider’s viscoelastic tissues is upper-bounded as $\left|P\left(z\right)\right|\leq c_{P1}+c_{P2}\left|z_{2}\right|$ for all $z\in R^{2}$ , where c_P1_, c_P2_ > 0 are known constants.

Property 5. The torque due to viscous damping is upper-bounded as $\left|\tau_{b}\left(z_{2}\right)\right|\leq c_{b}\left|z_{2}\right|$ for all $z_{2}\in R$ , where c_b_ > 0 is a known constant.

Property 6. The torques generated by system disturbances are bounded so that $T_{d}=\left[-c_{d},c_{d}\right]$ , where c_d_ > 0 is a known constant.

Property 7. Due to physical limitations of the rider, the volitional muscle torque is bounded so that $T_{vol}=\left[-c_{vol},c_{vol}\right]$ , where c_vol_ > 0 is a known constant.

Property 8. For each m∈M , the muscle control effectiveness is upper-bounded so that $\left|g_{m}\left(z\right)\right|\leq {\bar{c}}_{m}$ for all $z\in R^{2}$ , where ${\bar{c}}_{m}>0$ is a known constant.

Property 9. The set-valued mapping $F_{u}:R^{2}\times U⇉R^{2}$ is outer semicontinuous, locally bounded, and convex-valued. These properties follow from continuity of the defining functions and from Properties 6 and 7.

## 3 Control Design

**FIGURE 1:**
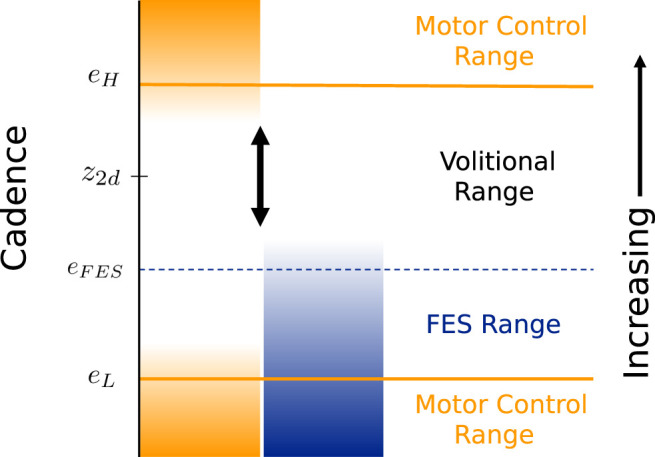
Illustration of the regions of applied control effort as a function of cadence. No control effort is applied in the volitional range near the setpoint z_2d_. The electric motor control input increases when the cadence approaches the boundaries defined by e_H_ and e_L_, and the FES control signal does the same near e_FES_. The size of each region is adjustable. The cadence range between e_H_ and e_L_ is rendered asymptotically stable by the developed control system.

Figure 1 shows a schematic of the staggered control regions for the developed system. The volitional range is a region near the setpoint z_2d_ where the control inputs are zero, thereby forcing the rider to pedal on their own volition (optionally, a nominal amount of assistance or resistance can be provided based on the needs of the rider). When the cadence is slower than z_2d_, FES assistance is provided before assistance from the electric motor. When the cadence is faster than the setpoint, only the electric motor is used because creating resistive torque with FES by stimulating antagonistic muscles is undesirable. The rider or clinician can modify the amount of control effort provided and the size of the controlled regions using parameters adjusted for the specific individual.

### 3.1 Control Objective

To formalize the control objective, we define the tracking error e as the deviation of the cadence state z_2_ from a constant setpoint z_2d_ > 0,^3^

$$e≜z_{2}-z_{2d}.$$ (5)

The primary control objective is to guarantee a safe and effective therapy by constraining the rider’s cadence to the safe set $S≜\left\{z\in R^{2}:e_{L}\leq e\leq e_{H}\right\}$ , where e_L_ < 0 < e_H_ are user-defined constants. The FES control inputs attempt to constrain the rider’s cadence to the secondary set $S_{FES}≜\left\{z\in R^{2}:e\geq e_{FES}\right\}$ . Because the FES inputs are only intermittently available and must be less than the comfort threshold $\bar{u}$ , the cadence may not remain within $S_{FES}$ . However, this construction is useful for design purposes. To ensure that FES stimulation is active before torque is added by the electric motor, the design specifies that e_L_ < e_FES_ < 0.

The goal is to synthesize, in a systematic way, controllers that render a given set of states uniformly globally asymptotically stable (UGAS) while using the minimum required effort inside the set of interest.^4^ Combining ideas from CLF theory with recent developments regarding CBFs, this task is accomplished by using a QP to enforce a constraint on the control input that is induced by a candidate barrier function. The following lemma, presented in a more generic form than in our preliminary work in Isaly et al. (2020), gives conditions under which a QP-based control law with a single constraint is feasible and locally Lipschitz continuous. The closed-form solution of the QP in the absence of a nominal controller (but including the case of multiple control inputs) has also been presented in Freeman and Kokotovic (1996, Section 4.2.2) and was developed in detail in Xu et al. (2015), Thm. 8. In those works, the feasibility condition was guaranteed by assuming the existence of a CLF or CBF, respectively. Because the addition of a nominal controller is a minor extension of the available literature, we do not present a proof of the result. Lemma 1 applies to the control laws developed in the subsequent sections.

Lemma 1. Let the functions $a:R^{n}\to R$ , $b:R^{n}\to R$ , and $u_{nom}:R^{n}\to R$ be locally Lipschitz on $R^{n}$ and satisfy the following feasibility condition:

$$a\left(z\right)=0\Rightarrow b\left(z\right)<0.$$ (6)

Then the set-valued mapping $\bar{U}:R^{n}⇉R$ defined by $\bar{U}\left(z\right)≜\left\{u\in R:a\left(z\right)u+b\left(z\right)\leq 0\right\}$ is non-empty for all $z\in R^{n}$ and the controller

$$\begin{array}{ll} u^{*}\left(z\right)≜\underset{u\in R}{\text{arg min}}\; & {\left|u-u_{nom}\left(z\right)\right|}^{2}\\ s.t. & a\left(z\right)u+b\left(z\right)\leq 0 \end{array}$$ (7)

is locally Lipschitz on $R^{n}$ , and, for any point z^∗^ such that $a\left(z^{*}\right)=0$ , there exists a neighborhood $N\left(z^{*}\right)$ such that $u^{*}\left(z\right)=u_{nom}\left(z\right)$ for all $z\in N\left(z^{*}\right)$ . Furthermore, the controller in Eq. 7 has a closed-form solution given by

$$u^{*}\left(z\right)=\left\{\begin{array}{cc} -\frac{b\left(z\right)}{a\left(z\right)}\; & a\left(z\right)u_{nom}\left(z\right)+b\left(z\right)>0\\ u_{nom}\left(z\right)\; & otherwise. \end{array}\right.$$ (8)

Note that there is no division by zero in Eq. 8 since Eq. 6 implies that $u^{*}\left(z\right)=u_{nom}\left(z\right)$ when $a\left(z\right)=0$ . Also note that the claim regarding the neighborhood about points for which $a\left(z^{*}\right)=0$ does not hold in general if the inequality in Eq. 6 is changed to $b\left(z\right)\leq 0$ .

### 3.2 Motor Control Design

In this section, the electric motor control input is designed to ensure that the safe set $S=\left\{z\in R^{2}:e_{L}\leq e\leq e_{H}\right\}$ is UGAS for a given z_2d_ > 0. Our development is based on the design procedure described in Isaly et al. (2020) and the theoretical results for hybrid systems in Maghenem and Sanfelice (2021) and Goebel et al. (2012), where Maghenem and Sanfelice (2021) considers barrier functions specifically. The safe set S is encoded by the barrier function candidate $B_{e}:R^{2}\to R$ defined as

$$B_{e}\left(z\right)≜\frac{1}{2}M\left(z_{1}\right)\left(\frac{e^{2}}{\beta \left(e\right)}-1\right)$$ (9)

where

$$\beta \left(e\right)≜\left\{\begin{array}{cc} e_{L}^{2}\; & e\leq 0\\ e_{H}^{2}\; & e>0. \end{array}\right.$$

Equivalent to the original definition, we have $S=\left\{z\in R^{2}:B_{e}\left(z\right)\leq 0\right\}$ . The barrier function is designed to be continuously differentiable while encoding the potentially asymmetric (about e = 0) boundary of the set S .

While barrier functions are typically associated with forward invariance, they are naturally extensible to enforcing the stronger property of asymptotic stability. Asymptotic stability is beneficial for real-world applications since it guarantees robustness to perturbations from the safe set. For continuous-time systems, the stronger asymptotic stability condition is embodied in the definition of a zeroing CBF (ZCBF) Xu et al. (2015). Inspired by the ZCBF approach in our preliminary work in Isaly et al. (2020) and the work regarding CLFs for hybrid systems in Sanfelice (2013), we constrain the control input according to the so-called regulation map $\tilde{U}:R^{2}⇉U$ defined as

$$\tilde{U}\left(z\right)≜\left\{u\in U:\left\langle \nabla B_{e}\left(z\right),f\right\rangle \leq -\gamma_{e}\left(e\right),\;\forall f\in F_{u}\left(z,u\right)\right\},$$ (10)

where

$$\gamma_{e}\left(e\right)≜k_{b1}\left(\frac{e^{2}}{\beta \left(e\right)}-1\right),$$ (11)

and k_b1_ > 0 is a control gain. In the stability analysis of Section 4, we show that selecting a continuous controller from $\tilde{U}$ ensures that B_e_ acts as a Lyapunov function for the closed-loop dynamics outside the set S . In particular, the condition in Eq. 10 will be used to apply Proposition 3.27 of Goebel et al. (2012) to conclude that S , redefined to include some additional states, is UGAS.

Remark 2. The definition of a ZCBF requires that $\gamma_{e}\left(z\right)≜\alpha \left(B_{e}\left(z\right)\right)$ for an extended class K function *a* Xu et al. (2015). However, requiring dependence of γ_e_ on the barrier function candidate can be restrictive, and is not necessary to obtain theoretical guarantees. Because the value of the function M in B_e_ is unknown and cannot be implemented in a control law, we use γ_e_ instead of the typical ZCBF-based selection when defining $\tilde{U}$ in Eq. 10. For both ZCBFs and the choice of γ_e_ in Eq. 11, the mapping in Eq. 10 enforces asymptotically stabilizing conditions on the barrier function at states outside the safe set.

The regulation map in Eq. 10 is not directly useful for design purposes because uncertainty in the dynamics prevents computation of the inequality (i.e., constraint) used to define $\tilde{U}$ . In the rest of this section, we develop a new regulation map based on a computable constraint that is implementable in a QP like Eq. 7. The resulting QP-based controller is a locally Lipschitz selection of $\tilde{U}$ . To compensate for the uncertainty introduced in Eq. 10 by F_u_, we employ Lyapunov-based robust control techniques to develop a worst-case upper bound of the inner product in Eq. 10. For any $\left(z,u\right)\in C_{u}$ and $f\in F_{u}\left(z,u\right)$ ,

$$\begin{array}{ll} \left\langle \nabla B_{e}\left(z\right),f\right\rangle & \in \frac{1}{2}\nabla M\left(z_{1}\right)z_{2}\left(\frac{e^{2}}{\beta \left(e\right)}-1\right)\\ & +\frac{1}{\beta \left(e\right)}e\left(\tau_{u}\left(z,u\right)-\tau_{F}\left(z\right)\right). \end{array}$$ (12)

The product $\frac{1}{\beta \left(e\right)}e\tau_{F}\left(z\right)$ contains the term $\frac{1}{\beta \left(e\right)}eV_{p}\left(z\right)z_{2}=\frac{1}{\beta \left(e\right)}e\left(V_{p}\left(z\right)e+V_{p}\left(z\right)z_{2d}\right)$ , leading to a cancellation with the term $\frac{1}{2}\nabla M\left(z_{1}\right)z_{2}\left(\frac{e^{2}}{\beta \left(e\right)}\right)$ since $\frac{1}{2}\nabla M\left(z_{1}\right)z_{2}=V_{p}\left(z\right)$ . Using Properties 2-7, it can then be shown that the unknown terms in Eq. 12 are upper bounded, for some constants c_1_ − c_3_, as

$$\left\langle \nabla B_{e}\left(z\right),f\right\rangle \leq C_{e}\left(e\right)+\frac{1}{\beta \left(e\right)}e\tau_{u}\left(z,u\right),$$ (13)

for each $\left(z,u\right)\in C_{u}$ and each $f\in F_{u}\left(z,u\right)$ , where

$$C_{e}\left(e\right)≜c_{1}+c_{2}\left|e\right|+c_{3}e^{2}.$$

In Eq. 13, the function τ_u_ depends on the motor control input u_e_ and the muscle control inputs u_M_. Because the value of the subsequently designed muscle control input will jump at discrete instances, it is desirable to decouple the motor input from the muscle input to ensure the continuity of the motor controller. A continuous motor controller will be more predictable and comfortable for the rider. Using Property 8, there exists a constant c_M_ > 0 such that

$$\begin{array}{ll} \frac{1}{\beta \left(e\right)}e\tau_{u}\left(z,u\right)=\frac{1}{\beta \left(e\right)}e & \left(c_{e}u_{e}+\tau_{FES}\left(z,u_{M}\right)\right)\\ & \leq \frac{c_{e}}{\beta \left(e\right)}eu_{e}+c_{M}\left|e\right|, \end{array}$$ (14)

for all $z\in R^{2}$ , $u_{e}\in R$ , and $u_{M}\in U_{M}$ . Using the definition of $U_{M}$ in Section 2, Eq. 14 applies to any muscle controller for which the inputs are bounded by the positive constant $\bar{u}$ . One can then define

$$K_{e}\left(e\right)≜k_{1}+k_{2}\left|e\right|+k_{3}e^{2}.$$ (15)

Selecting^5^

$$k_{1}\geq c_{1},\;k_{2}\geq c_{2}+c_{M},\;k_{3}\geq c_{3},$$ (16)

implies that

$$C_{e}\left(e\right)+\frac{1}{\beta \left(e\right)}e\tau_{u}\left(z,u\right)\leq K_{e}\left(e\right)+\frac{c_{e}}{\beta \left(e\right)}eu_{e}$$ (17)

for all $z\in R^{2}$ , $u_{e}\in R$ , and $u_{M}\in U_{M}$ . Since Eq. 17 is an upper bound for Eq. 13, we define a new regulation map ${\bar{U}}_{e}:R⇉R$ as

$${\bar{U}}_{e}\left(e\right)≜\left\{u_{e}\in R:K_{e}\left(e\right)+\frac{c_{e}}{\beta \left(e\right)}eu_{e}\leq -\gamma_{e}\left(e\right)\right\}.$$ (18)

The following result summarizes the preceding development and explains the utility of ${\bar{U}}_{e}$ .

Proposition 1. Assume k_1_, k_2_, and k_3_ satisfy the gain conditions in Eq. 16 and z_2d_ > 0. Then, for any $z\in R^{2}$ and $u_{M}\in U_{M}$ , if $u_{e}\in {\bar{U}}_{e}\left(e\right)$ , it follows that $\left(u_{e},u_{M}\right)\in \tilde{U}\left(z\right)$ .

The constraint used to define ${\bar{U}}_{e}$ in Eq. 18 can be written in the generic form of Lemma 1. Additionally, since the terms in Eq. 18 are no longer uncertain, the constraint can be enforced with the following implementable QP:

$$\begin{array}{ll} u_{e}^{*}\left(e\right) & ≜\underset{u_{e}\in R}{\mathrm{arg min}}\;{\left|u_{e}-u_{e}^{nom}\left(e\right)\right|}^{2}\\ & s.t.\;\;K_{e}\left(e\right)+\frac{c_{e}}{\beta \left(e\right)}eu_{e}\leq -\gamma_{e}\left(e\right), \end{array}$$ (19)

where $u_{e}^{nom}:R\to R$ is any locally Lipschitz nominal controller. According to Lemma 1, with $b\left(z\right)=K_{e}\left(e\right)+\gamma_{e}\left(e\right)$ , the controller is feasible if e = 0 implies that

$$K_{e}\left(0\right)+\gamma_{e}\left(0\right)=k_{1}-k_{b1}<0.$$ (20)

Since the parameters in Eq. 20 are user-selected, they can be designed to ensure the inequality holds. Given this gain condition, the controller has the properties described in Lemma 1, in particular, it is continuous. The closed-form solution to Eq. 19 can be developed from Eq. 8. The controller is implementable in either form but the closed-form solution is computationally faster and does not require an optimization package. Note that the piecewise linear function $e\;\;\mapsto \left(c_{e}/\beta \left(e\right)\right)e$ is locally Lipschitz, from which we derive local Lipschitz continuity of the controller.

Remark 3. During the experiments in Section 5, we investigated constant nominal controllers $u_{e}^{nom}≜u_{0}$ for some $u_{0}\in R$ . When u_0_ = 0, the controller is a minimum norm controller and the motor is off whenever possible while still ensuring safety. When the rider requires additional assistance, selecting a positive u_0_ leads to the motor being biased to assist, while a negative u_0_ leads to additional resistance and a more challenging training program. However, there is no theoretical obstacle to using a more complex nominal controller (e.g., one that tracks power).

### 3.3 Functional Electrical Stimulation Design

To describe the FES control input, we define a concatenated state vector $x≜\left(z,\sigma_{M},\tau \right)\in X$ , where $\sigma_{M}\in {\left\{0,1\right\}}^{6}$ is a vector of switching signals $\sigma_{m}\in \left\{0,1\right\}$ defined for each muscle m∈M , τ∈R is a timer variable, and $X≜R^{2}\times {\left\{0,1\right\}}^{6}\times R$ is the state space. The stimulation input to the rider’s muscle groups $u_{M}^{*}:X\;\;\to R^{6}$ is defined for each muscle as^6^

$$u_{m}^{*}\left(x\right)≜{\text{sat}}_{\bar{u}}^{+}\left(\sigma_{m}u_{FES}^{*}\left(e\right)\right),$$ (21)

where $u_{FES}^{*}:R\to R$ will be defined subsequently. The switching signals are updated at jumps according to the rule

$$\sigma_{m}^{+}=\left\{\begin{array}{cc} 1\; & z_{1}\in Q_{m}\\ 0\; & z_{1}\notin Q_{m}, \end{array}\right.$$ (22)

where $Q_{m}$ was defined in Section 2. The update rule specifies that the rider’s muscles are stimulated in regions where they are able to produce positive torque. The rule for σ_m_ and the timer variable τ will be used to define a hybrid system in Section 4.

As discussed in Section 3.1, it is generally not possible to maintain the cadence in the set $S_{FES}=\left\{z\in R^{2}:e\geq e_{FES}\right\}$ because the stimulation input $u_{m}^{*}$ is limited in magnitude and only intermittently available. The function $u_{FES}^{*}$ in Eq. 21 represents a selection of the input that would render the set $S_{FES}$ asymptotically stable in the absence of these obstacles (and subject to some gain conditions). The development of $u_{FES}^{*}$ is very similar to the one in Section 3.2 and is therefore omitted to avoid redundancy. Moreover, we do not make claims in the forthcoming stability analysis regarding $S_{FES}$ . The FES input is defined by the following QP:

$$\begin{array}{ll} u_{FES}^{*}\left(e\right) & ≜\underset{u_{FES}\in R}{\mathrm{arg min}}\;\left|u_{FES}-u_{FES}^{nom}\left(e\right)\right|\\ & s.t.\;\;K_{FES}\left(e\right)+\frac{1}{\beta_{2}\left(e\right)}eu_{FES}\leq -\gamma_{FES}\left(e\right). \end{array}$$ (23)

where $u_{FES}^{nom}:\;\;R\to \;\;R$ is any locally Lipschitz nominal controller,

$$K_{FES}\left(e\right)≜k_{4}+k_{5}\left|e\right|+k_{6}e^{2},$$

$$\gamma_{FES}\left(e\right)≜k_{b2}\left(\frac{e^{2}}{\beta_{2}\left(e\right)}-1\right),$$

and

$$\beta_{2}\left(e\right)≜\left\{\begin{array}{cc} e_{FES}^{2}\; & e\leq 0\\ e_{H}^{2}\; & e>0. \end{array}\right.$$

When e = 0, the feasibility condition (6) in Lemma 1 requires that

$$K_{FES}\left(0\right)+\gamma_{FES}\left(0\right)=k_{4}-k_{b2}<0,$$ (24)

under which the function $u_{FES}^{*}$ has the properties described in the lemma. Similar to the previous section, the parameters in Eq. 24 are user-selected and can be designed to ensure the inequality holds.

When the nominal controller in Eq. 23 is set to $u_{FES}^{nom}≜0$ , then from the closed-form solution in Eq. 8 it can be determined that $u_{FES}^{*}\left(e\right)\leq 0$ for all e ≥ 0. In this case, because of the use of the saturation function in Eq. 21, it follows that $u_{m}^{*}\left(x\right)=0$ for all x∈X such that e ≥ 0. The inclusion of a nominal controller in the QP defining $u_{FES}^{*}$ gives the operator flexibility to provide stimulation at points where e ≥ 0. When using a nominal controller with $u_{FES}^{nom}\left(e\right)>0$ for e ≥ 0, FES stimulation produces torque to increase the cadence above the setpoint z_2d_. However, it is always the case that $u_{FES}^{*}\left(e\right)\to 0$ as e → e_H_. By combining nominal assistance from FES with nominal resistance from the electric motor, a more intense training program can be designed where the rider must work against resistive torque from the electric motor to stay near the setpoint. We provide experimental results for a higher intensity configuration of the control system in Section 5.

## 4 Stability Analysis

We model the closed-loop system as a hybrid system $H=\left(C,F,D,G\right)$ with state $x=\left(z,\sigma_{M},\tau \right)\in X$ , where the state space X was defined in Section 3.3. The hybrid system will periodically update the muscle switching signals σ_m_ according to the rule in Eq. 22. The timer variable τ increases at a constant rate until reaching a dwell-time τ_D_ > 0, at which point a jump occurs and each signal in σ_M_ is updated. Governing jumps by a dwell-time prevents multiple jumps from occurring in the same time instant, and models a computational implementation of the switching signals, where the values of the logic variables would be updated periodically at a fixed sampling frequency. In practice, the dwell-time τ_D_ will be the sampling frequency.

The hybrid system H is defined as follows. The flow map F:X⇉X is

$$\left[\begin{array}{c} \dot{z}\\ {\dot{\sigma }}_{M}\\ \dot{\tau } \end{array}\right]\in \left[\begin{array}{c} F_{u}\left(z,u^{*}\left(x\right)\right)\\ 0\\ 1 \end{array}\right]≜F\left(x\right),$$

where $u^{*}\left(x\right)≜\left(u_{e}^{*}\left(e\right),u_{M}^{*}\left(x\right)\right)$ and F_u_ is defined in Eq. 2. The flow set is

$$C≜\left\{x\in X:\tau \in \left[0,\tau_{D}\right]\right\}.$$

Jumps occur when the timer state τ grows to τ_D_,

$$D≜\left\{x\in X:\tau =\tau_{D}\right\}.$$

The jump map G:X⇉X is defined component-wise as $G\left(x\right)≜\left(z,G_{\sigma }\left(x\right),0\right)$ , where the state z does not change at jumps (z^+^ = z), the timer τ resets to zero at jumps (τ^+^ = 0), and $G_{\sigma }:X⇉{\left\{0,1\right\}}^{6}$ is the outer semicontinuous Krasovskii regularization of the map in Eq. 22 Goebel et al. (2012), Def. 4.13. For each m∈M , the corresponding component of G_σ_ is equal to

$$G_{\sigma_{m}}\left(x\right)=\left\{\begin{array}{cc} 1\; & z_{1}\in Q_{m}\\ \left\{0,1\right\}\; & z_{1}\in \partial Q_{m}\\ 0\; & \text{otherwise.} \end{array}\right.$$ (25)

The set-valued case in Eq. 25 indicates that if τ reaches τ_D_ when the state z_1_ is precisely on the boundary of $Q_{m}$ , the signal σ_m_ may or may not jump. Performing such a regularization leads to some robustness properties due to the fact that H is a well-posed hybrid system Goebel et al. (2012, Ch. 6).

Remark 4. The gain conditions in Eqs. 20, 24 must be satisfied because they lead to the feasibility of the QP-based controllers. The conditions are restated here for emphasis:

$$k_{1}<k_{b1},\;k_{4}<k_{b2}.$$

Theorem 1. Consider the closed-loop cycle-rider system H . Assume the control gains satisfy the conditions in Eqs. 16, 20, 24, and z_2d_ > 0. Then the safe set $\tilde{S}≜\left\{x\in C\cup D:e_{L}\leq e\leq e_{H}\right\}$ is UGAS for H . Additionally, H is a well-posed hybrid system.

Proof. Since B_e_ in Eq. 9 is such that $\tilde{S}=\left\{x\in C\cup D:B_{e}\left(z\right)\leq 0\right\}$ , the function B_e_ is a valid barrier function candidate Maghenem and Sanfelice (2021), Def. 3. Using Lemma 1, the gain conditions in Eqs. 20, 24 guarantee that the controllers in Eqs. 19, 21, respectively, are feasible. By design, $u_{e}^{*}\left(e\right)\in {\bar{U}}_{e}\left(e\right)$ for all e∈R and $u_{m}^{*}\left(x\right)\in U_{M}$ for all x∈X . Proposition 1 then shows that $u^{*}\left(x\right)\in \tilde{U}\left(z\right)$ for all x∈X . It follows from the definition of $\tilde{U}$ in Eq. 10 that

$$\left\langle \nabla B_{e}\left(z\right),f\right\rangle \leq -\gamma_{e}\left(e\right),$$ (26)

for all x ∈ C and each $f\in F\left(x\right)$ . Moreover, the jump map is such that

$$B_{e}\left(z^{+}\right)=B_{e}\left(z\right),$$ (27)

for all x ∈ D and each $\left(z^{+},\sigma_{M}^{+},\tau^{+}\right)\in G\left(x\right)$ . The barrier function does not decrease at jumps, but there is sufficient flow time to guarantee an overall decrease along solutions. More specifically, the dwell-time τ_D_ ensures that for any solution ϕ to H , if $\left(t,j\right)\in \text{dom}\;\phi$ , then $t\geq \tau_{D}\left(j-1\right)$ . Thus, for any T ≥ 0 and $\left(t,j\right)\in \text{dom}\;\phi$ , if t + j ≥ T then $t\geq \left(\tau_{D}/\left(1+\tau_{D}\right)\right)\left(T-1\right)$ . We use this bound on the flow time to apply Proposition 3.27 of Goebel et al. (2012).

The conditions in Eqs. 26, 27, and the fact that $G\left(x\right)\subset C\cup D$ for all x ∈ D allow us to apply Theorem 1 of Maghenem and Sanfelice (2021) to conclude that the set $\tilde{S}$ is forward pre-invariant for H . Furthermore, the barrier function B_e_ is a Lyapunov function for the restricted hybrid system $H_{r}=\left(C_{r},F,D_{r},G\right)$ with $C_{r}≜C\cap I$ and $D_{r}≜D\cap I$ , where $I≜\left\{x\in X:B_{e}\left(z\right)\geq 0\right\}$ , which is the restriction of H to the zero superlevel set of B_e_. It can be shown that there exists^7^ a continuous, positive definite function $\rho :R_{\geq 0}\to R_{\geq 0}$ such that $\rho \left({\left|x\right|}_{\tilde{S}}\right)\leq \gamma_{e}\left(e\right)$ for all x ∈ C_r_. Using Property 1, there are class $K_{\infty }$ functions^8^ α_1_ and α_2_ such that $\alpha_{1}\left({\left|x\right|}_{\tilde{S}}\right)\leq B\left(z\right)\leq \alpha_{2}\left({\left|x\right|}_{\tilde{S}}\right)$ for all $x\in C_{r}\cup D_{r}\cup G\left(D_{r}\right)$ . Thus, Proposition 3.27 of Goebel et al. (2012) can be applied to conclude that $\tilde{S}$ is uniformly globally pre-asymptotically stable (UGpAS) for $H_{r}$ Goebel et al. (2012), Def. 3.6. That $\tilde{S}$ is UGpAS for the unrestricted system H follows from forward pre-invariance of $\tilde{S}$ for H , since for any solution ϕ to H , if $\phi \left(t,j\right)\in \tilde{S}$ then ${\left|\phi \left(t^{\prime },j^{\prime }\right)\right|}_{\tilde{S}}=0$ for all $\left(t^{\prime },j^{\prime }\right)\in \text{dom}\;\phi$ with t′ ≥ t, j′ ≥ j. Therefore, solutions to $H_{r}$ that terminate on the boundary of $\tilde{S}$ can be extended as solutions to H that remain in $\tilde{S}$ .

To conclude that $\tilde{S}$ is UGAS^9^, it remains to show that each maximal solution to H is complete. Towards this end, we invoke Proposition 6.10 of Goebel et al. (2012). We first note that the dynamics satisfy the hybrid basic conditions Goebel et al. (2012), Asm. 6.5 because C and D are closed; G is outer semicontinuous and locally bounded; and F is outer semicontinuous, locally bounded, and convex-valued by Property 9 and Lemma 3.2 in Sanfelice (2013). It follows that H is a well-posed hybrid system Goebel et al. (2012), Thm. 6.30. Next, every point x ∈ ∂C\D has the component τ = 0. The fact that $\dot{\tau }=1$ implies that $F\left(x\right)\cap T_{C}\left(x\right)\neq \emptyset$ at any x ∈ ∂C\D, where $T_{C}\left(x\right)$ is the tangent cone to C at x. It is then straightforward to conclude that the condition (VC) in Proposition 6.10 holds for all x ∈ C\D. Moreover, $G\left(D\right)\subset C\cup D$ . Thus, Proposition 6.10 shows that a maximal solution is either complete or escapes in finite time by flowing.

To eliminate the possibility that maximal solutions escape in finite time by flowing, we first let ϕ be a solution to H . From the definition of UGpAS in Definition 3.6 of Goebel et al. (2012), the distance of ϕ from $\tilde{S}$ is bounded. From the definition of $\tilde{S}$ , the component of ϕ corresponding to the state e is bounded. Using this information, we conclude from continuity of $e\;\;\mapsto \;u_{e}^{*}\left(e\right)$ and the use of the saturation function in the definition of $u_{M}^{*}$ in Eq. 21 that for the concatenated controller u*, the set $u^{*}\left(\text{rge}\;\phi \right)$ is bounded, where $\text{rge}\;\phi ≜\left\{\phi \left(t,j\right):\left(t,j\right)\in \text{dom}\;\phi \right\}$ . Then, from boundedness of the e component of ϕ and Properties 2-7, it can be shown that the set $F\left(\text{rge}\;\phi \right)$ is bounded. It follows that solutions do not terminate in finite time by flowing (cf. Kamalapurkar et al. (2020)). Thus, each maximal solution to H is complete, and $\tilde{S}$ is UGAS for H . ■

## 5 Experimental Results

The developed barrier function controller was tested on five participants and compared against uncontrolled volitional pedaling and the 3-Mode (3M) controller developed in Rouse et al. (2020), Section 3. As described in Section 1, the main idea behind the 3M controller is to create a region near the setpoint z_2d_ where no assistance is provided, with discontinuous control effort being applied on the boundary of the region. In contrast to the barrier function controller, the electric motor controller for the 3M controller is coupled with FES stimulation via the angular position state z_1_, so that the motor is inactive whenever FES is active, and vice versa.

The barrier function controller can be configured for various purposes based on the needs of the rider. Generally, there is a trade-off where smaller user-defined cadence ranges lead to greater applied control effort. Protocol A was designed to investigate whether the controller can reduce the variance in the rider’s cadence by constraining their cadence within a small range. Such a trial provides a point of comparison with the 3M controller and uncontrolled volitional pedaling and generates data where the controller is more active. Protocol B was designed to show how assistance from the motor can be reduced by selecting a wider safe range, thereby encouraging more volitional contributions from the rider. In fact, Protocol B featured a nominal amount of resistance from the motor, making the program more challenging and requiring additional power output from the rider.

Due to COVID-19 related difficulties in scheduling participants with neurological conditions, the trials for this demonstration were done with able-bodied subjects. Each participant gave written informed consent approved by the University of Florida Institutional Review Board (IRB201600881). Participants 1-3 were male, participants 4 and 5 were female, and all ranged in age from 21–29 years old.

### 5.1 Testbed

The experimental testbed consisted of a stationary recumbent tricycle (TerraTrike Rover) with a 250 W, 24 V motor (Unite Motor Co.) coupled to the drive chain as described in Bellman et al. (2017), Section 5.1. To measure position and cadence, an optical encoder with an angular resolution of 20,000 pulses per revolution (US Digital H1) was mounted to the crank using spur gears. The motor was actuated using an Advanced Motion Controls^10^ motor driver and current-controlled power supply. Stimulation was delivered to the rider’s quadricep, hamstring, and gluteal muscle groups via self-adhesive electrodes provided compliments of Axelgaard Manufacturing Co., Ltd. A current-controlled stimulator (Hasomed Rehastim) delivered symmetric, rectangular, and bi-phasic pulses at fixed amplitude (90 mA, 80 mA, and 70 mA for the quadriceps, hamstrings, and gluteals, respectively) and frequency (60 Hz), while the pulse width was used as the control input. A desktop computer running real-time control software (QUARC integrated with Simulink) was used to interface the controllers and hardware through a data acquisition board (Quanser QPIDe) with a sampling rate of 1,000 Hz. For additional safety, an emergency stop switch was mounted on the cycle to allow the participant to end the experiment if required.

### 5.2 Procedure

The primary testing procedure (Protocol A) consisted of 180-s tests for each of the three configurations (barrier function, 3M, and volition-only) under consideration. The volitional pedaling trial was always first, followed by a random selection of either the barrier function or 3M controller. The riders were asked to track a setpoint of z_2d_ = 50 RPM. The safe set boundary for the barrier function controller was encoded by e_L_ = − 5 RPM and e_H_ = 5 RPM, while e_FES_ = − 3 RPM. The inactive region for the 3M controller was 48–52 RPM, which is comparable to the range 50–55 RPM that was used for the experiments in Rouse et al. (2020). The rider was shown a live plot of their cadence featuring a visible indication of the setpoint. Due to differences between the barrier function and 3M controllers, participants were not shown the boundaries of the safe set^11^.

The 180 s tests started with a 20 s ramp-up phase where the rider sat passively while the motor brought their cadence to the setpoint. To ensure that the presented data represented steady-state operation, the ramp-up and an additional 20 s after it was excluded from each dataset in post-processing. For each configuration, there was a separate warm-up run before the recorded session so the rider could become accustomed to the controller.

Measurements of the position of the rider’s legs with respect to the cycle were used to determine the regions of effective torque transfer $Q_{m}$ for each muscle (see Bellman et al. (2017), Section 5.2) for more details). The cycle was initially operated at 50 RPM and open-loop stimulation was applied to one muscle group at a time to determine the comfort limit $\bar{u}$ for each muscle. The FES inputs are scaled by the comfort threshold in addition to being saturated^12^. The nominal controllers were $u_{e}^{nom}=u_{FES}^{nom}=0$ . The control gains were adjusted by plotting the control inputs as a function of the cadence error, which produced a visualization of the regions of applied control effort. Small adjustments to the gains were made for each participant based on their preferences during the warm-up run, which is a cause for variation in the data between participants. Some detailed discussion about the effect of the barrier function controller gains on performance is provided in Isaly et al. (2020) (see Remark 1 and Section 5.4).

Additional trials were conducted to highlight unique aspects of the barrier function controller, which were performed with only one participant. Protocol B was designed to prioritize power output from the rider over cadence tracking. For this alternative trial, the width of the safe range was larger, with e_L_ = − 12 RPM, e_H_ = 10 RPM, and e_FES_ = − 6 RPM. Because the rider (Participant #2) was able-bodied, we chose to make the program more challenging by adding nominal controllers with $u_{e}^{nom}<0$ and $u_{FES}^{nom}>0$ , which means that near the setpoint the electric motor produced resistive torque while FES provided assistance. The boundaries e_L_ and e_H_ were displayed on-screen for this trial. In Protocol C, Participant #1 was asked to provide no volitional effort for both the 3M and barrier function controllers (Figure 4). Because of problems with the 3M controller for this trial, we did not proceed with testing the no-volition configuration on other participants.

### 5.3 Results

**FIGURE 2:**
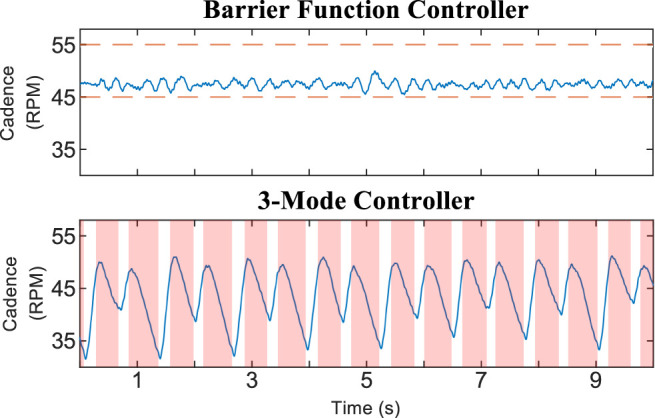
Cadence and motor control input for 30-s segments of the trials using Protocol A. Random selection was used to choose which participants and time periods were plotted. The time axis is offset to zero for readability. The dashed orange line in the barrier function cadence plot indicates the boundary of the safe set S , which was not meaningful for the other two configurations.

**FIGURE 3:**
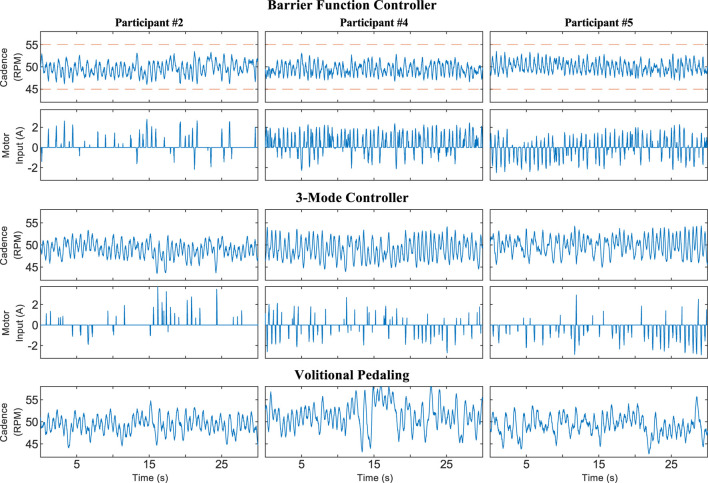
Zoomed view of a 5-s time period during a trial using Protocol A showing the cadence, motor control input, and stimulation pulse-width (PW) for Participant 5. Random selection was used to choose which participant and time period were plotted. The time axis is offset to zero for readability. A pulse-width feedforward term of 10 μs was used to facilitate stimulation. The stimulation does not significantly affect the participant at or below 10 μs.

Table 1 shows relevant statistics for the three configurations tested for Protocol A, including the average and standard deviation of the cadence, percentage of the trial duration for which FES was actively stimulating, and time spent outside of the safe set S . Two integrals of the electric motor input are given to distinguish resistive torque, for which the rider must pedal harder to compensate, and assistive torque, which implies work done by the motor and not the rider. The barrier function controller produced the lowest standard deviation in cadence for each participant and led to greater FES usage, but generally used more assistive torque from the motor than the 3M controller. The 3M controller produced less assistive torque because the electric motor was off in regions of the crank cycle where FES was active. The discrete switching between motor and FES, along with switching on the boundary of the inactive zone, caused numerous discontinuities in the 3M motor controller; Table 1 shows an average of 493 switches per trial. For all participants, the riders’ cadence took values in the range 40.7–56.1 RPM for the 3M controller, 45.4–55.3 RPM for the barrier function controller, and 40.5–59.2 RPM for uncontrolled pedaling. The barrier function controller constrained each rider’s cadence to the user-defined range of 45–55 RPM for all but a negligible amount of time; an average of six sampled data points or approximately 0.004% of the trial duration. Segments of the trials for three randomly selected participants are shown in Figure 2. A zoomed view featuring the FES stimulation input for a single participant is shown in Figure 3.

**TABLE 1:** Protocol A: Cycling metrics during steady-state operation (140 second trial).

			Participant number				
Controller	Metric	Average	1	2	3	4	5
Barrier Function	Avg. Cad. [RPM]	49.97	49.82	49.59	50.85	49.31	50.26
	Cad. SD [RPM]	1.38	1.37	1.50	1.54	1.27	1.25
	Min/Max Cad. [RPM]	46.2/54.7	46.6/54.2	45.7/54.0	46.5/55.3	45.4/55.1	46.9/54.2
	$\int {\left(u_{e}^{*}\right)}^{+}\;dt$ (Assistive Torque) [A⋅s]^a^	24.82	18.64	15.83	2.79	67.72	19.12
	$\int {\left(u_{e}^{*}\right)}^{-}\;dt$ (Resistive Torque) [A⋅s]^a^	-20.84	-10.25	-3.93	-33.39	-13.63	-42.99
	FES Usage [% trial duration]^b^	27.33	39.34	33.61	15.77	31.77	16.18
	Time Outside S [s]^c^	0.006	0	0	0.02	0.01	0
3-Mode	Avg. Cad. [RPM]	49.66	49.73	48.87	50.79	48.94	49.98
	Cad. SD [RPM]	1.83	1.57	1.89	1.62	2.21	1.87
	Min/Max Cad. [RPM]	43.2/55.1	44.6/55.5	40.7/53.3	46.3/55.1	40.7/56.1	43.9/55.6
	$\int u_{e}^{+}\;dt$ (Assistive Torque) [A⋅s]^a^	5.87	1.73	14.4	0.22	10.49	2.52
	$\int u_{e}^{-}\;dt$ (Resistive Torque) [A⋅s]^a^	-20.32	-8.72	-3.41	-47.39	-16.98	-25.13
	FES Usage [% trial duration]^b^	17.04	12.16	23.42	3.03	31.56	15.05
	Num. Motor Switches^d^	493	335	414	530	636	549
Volitional	Avg. Cad. [RPM]	49.91	49.62	49.13	49.82	50.87	50.11
	Cad. SD [RPM]	2.13	2.13	1.81	1.76	2.72	2.21
	Min/Max Cad. [RPM]	42.0/56.0	42.4/55.2	40.5/54.8	43.3/54.3	42.0/59.2	41.6/56.6

a Indicates the postive or negative component of the integral, e.g., $\int u_{e}^{+}\;dt≜\int_{t_{0}}^{t_{f}}\max\left\{u_{e}\left(z\left(t\right)\right),0\right\}\;dt$ .

b Quantifies the percentage of the trial duration that FES was active at non-negligible pulse-width values greater than 10 μs.

c Computed as the number of recorded cadence values outside the set multiplied by the sampling time of 0.001 s.

d Quantifies the number of discontinuities in the motor control signal as a function of time.

**FIGURE 4:**
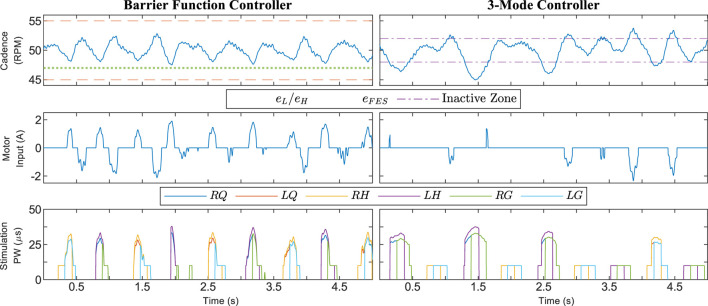
Cadence from a trial using Protocol C, where Participant #1 was asked to provide no volitional effort. The 3M controller was problematic in this scenario due to switching between FES and motor control. The shaded red regions in the 3-Mode plot correspond to times when the electric motor was switched off.

Figure 4 shows the trials using Protocol C, where Participant #1 provided no volitional effort. The barrier function controller was still able to keep the rider’s cadence within the safe set, while the 3M controller caused large oscillations; the mean ± SD cadence for the 3M controller was 43.74 ± 4.94 RPM. The cadence dropped as low as 31.54 RPM during the 3M trial because the electric motor was inactive in the shaded regions of Figure 4. When the motor was next switched on, it compensated using control action with a maximum magnitude of 17.74 A, which was relatively large compared to a maximum of 8.13 A for the 3M controller during the Protocol A trials.

**FIGURE 5:**
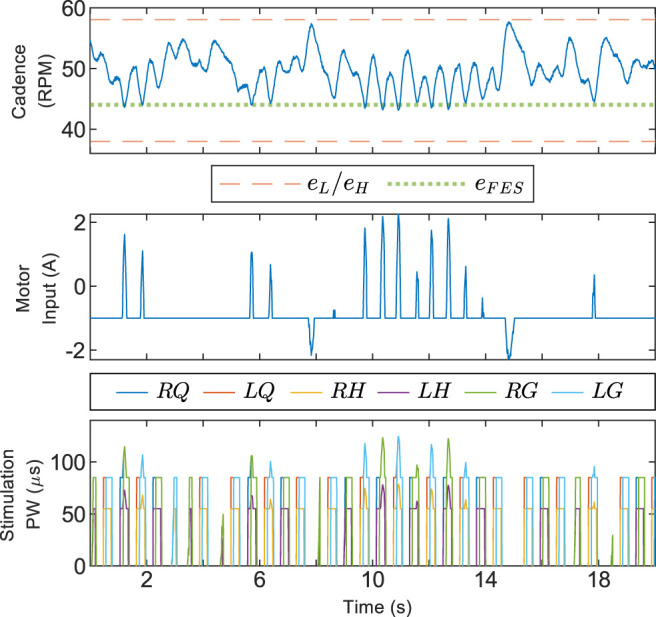
Segment of a trial using Protocol B, where alternative parameters for the barrier function controller were used. The safe set was much wider for this run, leading to less deviation of control inputs from the nominal value. Nonzero nominal inputs were used to intensify the training. The data was collected with Participant #2.

The results for Protocol B, where there were nonzero nominal controllers and a wider safe range, are displayed in Figure 5 and Table 2. There was high utilization of the electric motor to produce resistive torque, but low assistive torque production. The assistive torque was smaller than the Protocol A average and, in particular, was smaller than Participant #2’s results. The resistive torque was large and FES was more active for Protocol B due to the design of the nominal controllers. The electric motor deviated from its nominal value for only 7.7% of the trial duration and was providing assistance for 4.1% of the trial. The cadence standard deviation was higher than uncontrolled volitional pedaling, which was most likely because the control inputs were actively pushing the rider away from the setpoint. The assistive torque from the barrier function controller in Protocol B was comparable to the 3M controller in the Protocol A trials.

**TABLE 2:** Protocol B–barrier function controller: Cycling metrics during steady-state operation (140 second trial).

Metric^a^	Par. #2
Avg. Cad. [RPM]	49.87
Cad. SD [RPM]	2.80
Min/Max Cad. [RPM]	43.0/57.6
$\int {\left(u_{e}^{*}\right)}^{+}\;dt$ (Assistive Torque) [A⋅s]	4.76
$\int {\left(u_{e}^{*}\right)}^{-}\;dt$ (Resistive Torque) [A⋅s]	-119.01
FES on-time [%]	47.67
Time Outside S [s]	0

a See Table 1 for further descriptions of metrics.

### 5.4 Discussion

The results for Protocol A demonstrate that the barrier function controller can assist a rider in tracking the cadence setpoint while constraining their cadence within a user-defined range. Figure 3 shows that the control inputs ramp up before the cadence reaches the boundaries defined by e_H_, e_L_, and e_FES_, yet are inactive when the cadence is near the setpoint. Such a ramp-up is demonstrative of how barrier function methods can ensure a gradual transition from a nominal controller, which should be active on the interior of the safe set, to an invariance-ensuring controller on the boundary of the safe set. For Protocol A, the width of the nominal control range was fairly small, while the width was increased for Protocol B.

In some situations, increasing the training intensity for the rider will be preferred over improved cadence tracking. The relevant statistics are the assistive and resistive torques produced by the electric motor. Higher assistive torque indicates that less power is being produced by the rider. The barrier function controller is versatile and can be tuned to provide more or less interference from the control inputs. Figure 5 shows a trial using Protocol B where power output from the rider was prioritized by allowing larger cadence errors. The system’s operator can allow larger cadence errors by selecting a wider safe range, which also facilitates the design of a wider nominal control range. The use of nominal controllers for Protocol B intensified the training by providing more FES stimulation and resistance from the motor. The control inputs were at their nominal values for a significant portion of the experiment, validating that a wider safe range leads to less modification of the nominal inputs. The nominal inputs can alternatively be designed so there is less resistance from the motor or less control input overall. The fact that the assistive torque from the barrier function controller was comparable to the 3M controller suggests that staggering FES assistance before motor assistance, as in the barrier function controller, is a viable alternative to discrete switching between FES and motor control, as in the 3M controller.

A continuous motor controller is more comfortable for the rider. Continuity is the primary difference between the barrier function and 3M controller. The 3M controller is discontinuous whenever the cadence crosses the boundary of the inactive zone, or the angular position crosses the boundary of the FES stimulation regions $Q_{m}$ . The resulting large number of switches for the 3M motor controller is quantified in Table 1. A particular advantage of the motor being active in the $Q_{m}$ regions is that the barrier function controller is effective even when the rider produces little or no volitional effort, as shown in the trials using Protocol C (Figure 4). The 3M controller caused large oscillations in the rider’s cadence during Protocol C due to the discrete switching between FES and motor control. Since FES cannot always produce enough torque on its own, the cadence dropped in the FES stimulation regions, which was then met with large control effort from the motor upon exiting the region.

There were some small deviations from the safe set because the asymptotic stability of S is contingent on the selection of control gains, which must be large enough to compensate for the rider’s volitional effort and other dynamic effects. In practice, designing the gains to account for large volitional contributions from the rider leads to overly constraining the rider during normal operation. Designing controllers for asymptotic stability, rather than the weaker property of forward invariance, helps mitigate these effects. A property known as input-to-state stability, which is a consequence of the UGAS result in Theorem 1, can be interpreted as guaranteeing that some nearby set is asymptotically stable despite unaccounted-for disturbances of bounded magnitude Xu et al. (2015); Cai and Teel (2009). Thus, the control gains can be relaxed to favor comfortable and effective therapy while still ensuring that the cadence remains nearby the safe set.

## 6 Conclusion

This paper developed new FES and motor controllers that encourage the rider of a stationary cycle to provide volitional effort while constraining their cadence within a user-defined range. Using theoretical advances for barrier functions, the controllers are minimally invasive while transitioning gradually to a safety-ensuring controller on the boundary of the safe set. The control inputs are selected from regulation maps with sufficient regularity to ensure that optimal selections are locally Lipschitz functions of the cadence error. Robust control tools were used to develop the regulation maps, which are subsets of an original, uncertain map. The uniform global asymptotic stability of the user-defined safe set was certified with a hybrid system analysis. In the future, the performance of the controller can be improved by extending the development to a more complete dynamic model which accounts for muscle activation effects such as electromechanical delay in the rider’s muscles.

Experimental results showed that the control system improved the rider’s cadence tracking and effectively constrained their cadence within the safe set. The versatility of the controller was demonstrated with trials featuring two different objectives: improved cadence tracking or more power output from the rider. A significant next step is to perform more rigorous experiments on a set of participants with neurological conditions. These riders have a reduced ability to provide volitional contributions, so the results are expected to be significantly different from those of able-bodied riders, and would be of interest in more clinically focused literature.

## Data Availability

The raw data supporting the conclusions of this article will be made available by the authors, without undue reservation.

## Footnotes

1 For vectors $x\in R^{n}$ , $y\in R^{m}$ , $\left(x,y\right)≜{\left[x^{T},y^{T}\right]}^{T}$ .

2 The addition of a point a∈R and a set B⊂R is defined as $a+B≜\left\{a+b:b\in B\right\}$ .

3 Using the transformation in Eq. 5, we frequently use e in place of the cadence state z_2_.

4 A set that is UGAS is also forward invariant, meaning that trajectories starting inside the set remain in the set for all of time.

5 While the gain conditions in Eq. 16 will be needed to guarantee that the cadence is constrained to the set S , uniform global asymptotic stability ensures that the cadence remains nearby S even if the conditions do not hold (see Section 5.4).

6 The positive saturation function ${\text{sat}}_{\bar{u}}^{+}:R\to R$ is defined as ${\text{sat}}_{\bar{u}}^{+}\left(u\right)=0$ if u < 0, ${\text{sat}}_{\bar{u}}^{+}\left(u\right)=u$ if $u\in \left[0,\bar{u}\right]$ , and ${\text{sat}}_{\bar{u}}^{+}\left(u\right)=\bar{u}$ if $u>\bar{u}$ .

7 While more general techniques can be developed, it is sufficient here to define $\rho \left(s\right)≜\gamma_{e}\left(s+e_{H}\right)$ if $e_{H}\geq \left|e_{L}\right|$ or $\rho \left(s\right)≜\gamma_{e}\left(e_{L}-s\right)$ if $e_{H}<\left|e_{L}\right|$ .

8 A function $\alpha :R_{\geq 0}\to R_{\geq 0}$ is a class $K_{\infty }$ function if *a* is zero at zero, continuous, strictly increasing, and unbounded.

9 A set A is UGAS for a hybrid system H if it is UGpAS and every maximal solution to the system is complete, meaning the solution is defined on an unbounded hybrid time domain.

10 Advanced Motion Controls supported the development of the testbed by providing discounts on their branded items.

11 In the 3M controller, the user defines a region where no control is applied. An asymptotically stable region will be induced by the selection of the control gains, but it is not possible to compute this region explicitly. In the barrier function controller, the user defines the safe set boundary, while a computable region of no-control is induced by the selection of the control gains, and guaranteed to exist by Lemma 1.

12 Practical improvements like muscle-dependent control gains are not included in the theoretical development for simplicity.
